# Impairment of Muscle Function Causes Pupal Lethality in Flies Expressing the Mitochondrial Alternative Oxidase

**DOI:** 10.3390/biom15040570

**Published:** 2025-04-11

**Authors:** Carlos A. Couto-Lima, Sina Saari, Geovana S. Garcia, Gabriel H. Rocha, Johanna ten Hoeve, Eric Dufour, Marcos T. Oliveira

**Affiliations:** 1 Departamento de Biotecnologia, Faculdade de Ciências Agrárias e Veterinárias de Jaboticabal, Universidade Estadual Paulista “Júlio de Mesquita Filho”, Jaboticabal 14884-900, SP, Brazil; carlos.couto@unesp.br (C.A.C.-L.); geovana.garcia@unesp.br (G.S.G.); gabriel.hayashi@unesp.br (G.H.R.); 2 Faculty of Medicine and Health Technology, Tampere University, 33520 Tampere, Finland; 3 UCLA Metabolomics Center, Department of Molecular and Medical Pharmacology, University of California Los Angeles, Los Angeles, CA 90095, USA

**Keywords:** mitochondria, amino acid metabolism, nutrient deprivation, oxidative phosphorylation, musculature

## Abstract

The mitochondrial alternative oxidase (AOX) from the tunicate *Ciona intestinalis* has been explored as a potential therapeutic enzyme for human mitochondrial diseases, yet its systemic effects remain poorly understood. Here, we investigate the metabolic and physiological consequences of AOX expression during the development of *Drosophila* cultured under dietary stress. We show that the combination of strong, ubiquitous AOX expression and a low-nutrient condition leads to pupal lethality and severe defects in larval musculature, characterized by actin aggregation and muscle shortening. These structural abnormalities correlate with a decrease in larval biomass and motility. Interestingly, the muscle defects and the motility impairments vary in severity among individuals, predicting survival rates at the pupal stage. AOX expression in specific tissues (muscle, nervous system or fat body) does not individually recapitulate the lethal phenotype observed with ubiquitous expressions of the enzyme, indicating a complex metabolic imbalance. Metabolomic analysis revealed that the low-nutrient diet and AOX expression have opposite effects on most metabolites analyzed, especially in the levels of amino acids. Notably, supplementation of the low-nutrient diet with the essential amino acids methionine and/or tryptophan partially rescues pupal viability, body size, muscle morphology, and locomotion, whereas supplementation with proline and/or glutamate does not, highlighting a specific perturbation in amino acid metabolism rather than general bioenergetic depletion. These findings demonstrate that AOX expression disrupts metabolic homeostasis, with developmental and physiological consequences that must be considered when evaluating AOX for therapeutic applications.

## 1. Introduction

Although mitochondria have many important roles in eukaryotic cells, the bulk production of adenosine triphosphate (ATP) via oxidative phosphorylation (OXPHOS) is traditionally viewed as one of their main contributing processes for cellular function. Several mitochondrial inner membrane-embedded enzymes, such as nicotinamide adenine dinucleotide (NADH):ubiquinone oxidoreductase (complex I, CI), succinate dehydrogenase (complex II, CII), and mitochondrial glycerol-3-phosphate dehydrogenase (mGPDH), among others, may convergently initiate OXPHOS by catalyzing the reduction of coenzyme Q (CoQ/CoQH_2_). Because they re-oxidize key metabolites and electron carriers located in the mitochondrial matrix and outside mitochondria, these dehydrogenases may directly control the metabolism of nutrients such as monosaccharides, amino acids, and fatty acids. Starting a serial chain of redox reactions that we herein refer to as the cytochrome segment of the respiratory chain, the cytochrome *bc_1_* complex (complex III, CIII) oxidizes CoQH_2_, reducing cytochrome *c*, which in turn is re-oxidized by the cytochrome *c* oxidase (complex IV, CIV) in a reaction that uses molecular oxygen (O_2_) as the final electron acceptor, releasing water (H_2_O). This electron transfer system (ETS) formed by the aforementioned dehydrogenases combined with the cytochrome segment is paired with the pumping of protons from the mitochondrial matrix into the intermembrane space. The energy of the electrochemical gradient thus formed is eventually used by the ATP synthase complex to phosphorylate adenosine diphosphate (ADP), as protons return to the mitochondrial matrix [1,2].

The electrochemical gradient, the production of ATP, and the reoxidation of the intermediate electron carriers are crucial for the regulation of cellular metabolism. The OXPHOS function directly modulates the catabolic and anabolic functions of the tricarboxylic acid (TCA) cycle and may have various consequences depending on factors such as the tissue/cell type, stage of development, temperature and/or nutritional availability. For example, in proliferative tissues such as in tumors or in the growing *Drosophila* larva, a significant fraction of the TCA cycle intermediates serves as precursors for the synthesis of lipids, nucleotides, carbohydrates and proteins, in a process referred to as cataplerosis [3]. Although cataplerosis appears to be the most important function of mitochondria during biomass accumulation, it is strongly impaired if the reoxidation of NADH and/or CoQH_2_ by the ETS is compromised [4,5].

Protists, fungi, plants, and most metazoans, but not vertebrates or most insects, are endowed with a branched OXPHOS system involving a second terminal oxidase, the non-protonmotive alternative oxidase (AOX) [6]. AOX activity bypasses CIII/CIV, directly coupling O_2_ reduction with CoQH_2_ oxidation. Although AOX activity may decrease mitochondrial ATP synthesis, it can preserve electron flow and cellular redox potential and limit reactive oxygen species (ROS) production in the context of OXPHOS dysfunction (e.g., stress, overload or blockage) [5,7,8,9]. In humans, OXPHOS dysfunction is related to a variety of mitochondrial diseases, whose onset and severity vary broadly, with very limited effective treatments [10]. The premise that AOX activity would be beneficial for higher animals with OXPHOS dysfunction has been successfully tested in several models [11,12,13,14,15,16]. In addition to supporting respiration resistant to inhibitors of CIII and CIV, AOX enables the complete or partial recovery of deleterious phenotypes caused by mutations in subunits of the OXPHOS complexes [5,12,17,18,19] and is under consideration as a therapy enzyme [20,21].

Nevertheless, AOX expression studies in model organisms have been limited in regard to the alterations it may cause to normal metabolism and physiology, especially considering that AOX activity could partially uncouple mitochondria and generate excess heat. We have been using *Drosophila melanogaster* lines expressing AOX from the tunicate *Ciona intestinalis* (Ascidiacea) to assess possible limitations in the use of this enzyme in higher animals. AOX interference with cell signaling was demonstrated by the restoration of defective c-Jun N-terminal kinase (JNK) signaling during *Drosophila* development, through an as-yet unidentified mechanism [22]. We have also shown that AOX expression causes a dose-dependent depletion of mature spermatozoids, which leads to a dramatic disadvantage for AOX-expressing males in sperm competition assays [23]. Recently, we reported that AOX expression heavily compromises adult eclosion when the flies are cultured on a low-nutrient (LN), but not on standard laboratory (SD) diet [24]. Our SD diet contains 1.5% sucrose (*w*/*v*), 3% glucose, 1.5% maize flour, 1% wheat germ, 1% soy flour, 3% molasses, and 3.5% yeast extract, whereas, in the LN diet, the only source of nutrients is 3.5% yeast extract (*w*/*v*), which is normally sufficient for the *Drosophila* larvae to reach the adult stage. Structurally complex ingredients of the SD diet, such as maize flour, wheat germ or molasses rescued the lethality of AOX-expressing flies, but not simple compounds like sugars, vitamins, and minerals, nor additional yeast extract [25]. Mass spectrometry revealed a complex array of metabolites in a fraction of molasses that is rich in intermediates of the TCA cycle, among other molecules, but no compound that has been tested to date could individually rescue the lethality of AOX-expressing flies cultured on LN. This suggests that AOX may cause complex metabolic changes, which could explain why the transgene is unable to rescue deleterious phenotypes of some well-established mitochondrial mutants [26,27,28,29], and, yet, it was effective against genetic defects with no apparent direct link to mitochondria [22].

Here, we applied a metabolomic approach to gain more insight into the molecular basis of the lethal developmental interaction between AOX expression and LN diet. Our data suggest that AOX expression and the LN diet have opposite effects on larval amino acid metabolism, with severe consequences for larval muscle development. Pupal lethality was partially rescued by supplementing the feeding larvae with methionine or tryptophan, but not with proline or glutamate, suggesting that AOX disrupted specific metabolic processes crucial for fly development. The nature of these processes and their implications for tissue growth and AOX biology are explored further in the Discussion.

## 2. Materials and Methods

### 2.1. Fly Stocks and Diets

The *D. melanogaster* recipient line *w^1118^*, the driver lines *daughterless-GAL4* (*daGAL4*, BDSC ID# 55851) [30], *embryonic lethal abnormal visionC155-GAL4* (*elavGAL4*, ID# 458) [31], *myosin heavy chain-GAL4* (*mhcGAL4*, ID# 55133) [32] and *r-tetramer-GAL4* (*r4GAL4*, ID# 33832) [33], and the GAL4-dependent *UAS-GFP^Stinger^* line (ID# 84277) [34] were obtained from stock centers. Previously described transgenic lines carrying *UAS-AOX* constructs or the *UAS* promoter only (“empty” pUASTattB plasmid) were used: *UAS-AOX^F6^* (inserted into chromosome 2) [12], *UAS-AOX^8.1^* (chromosome 2), *UAS-AOX^7.1^* (chromosome 3), *UAS-mutAOX^2nd^* (chromosome 2), *UAS-mutAOX^3rd^* (chromosome 3) and *UAS-empty^2nd^* (chromosome 2) [18]. The double transgenic lines *UAS-AOX^8.1^;UAS-AOX^7.1^* and *UAS-mutAOX^2nd^;UAS-mutAOX^3rd^* were created by standard genetic crosses using lines carrying traditional balancer chromosomes (CyO and TM3, Sb). All fly lines were backcrossed with the recipient line *w^1118^* six-to-ten generations prior to the experiments. The fly lines were maintained on an SD diet [12], with a 12/12 h of a light/dark photoperiod cycle at 18 or 25 °C. The LN diet consisted of 3.5% yeast extract, 1% agar, and antimicrobials (0.1% niapigin and 0.5% propionic acid). Where indicated in figures and legends, diets were supplemented with the amino acids methionine, tryptophan, proline and glutamate at the concentrations shown.

### 2.2. Developmental and Larval Mobility Assays

**Figure 1:**
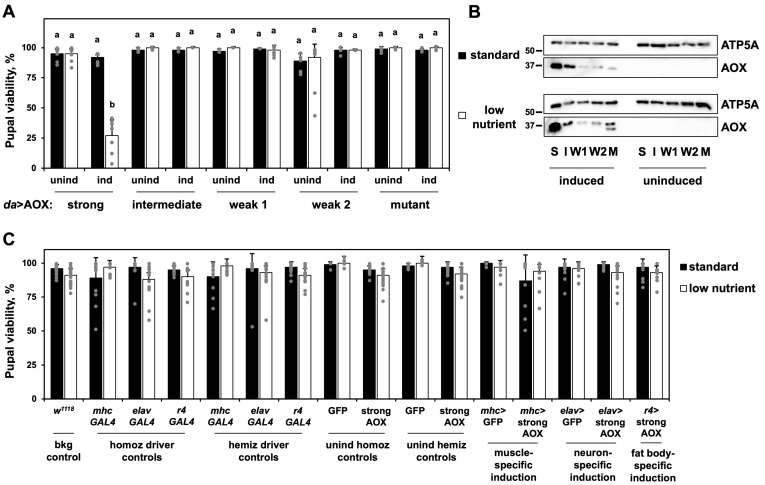
Pupal lethality is achieved via interaction between LN diet and strong ubiquitous expression of AOX: (**A**) Pupal viability (adult eclosion percentage) of flies with ubiquitous induction (ind) or not (unind) of AOX expression at the indicated levels. AOX expression is accomplished by crossing the homozygous *daGAL4* line with homozygous *UAS-AOX^F6^* [12] for strong levels, *UAS-AOX^8.1^; UAS-AOX^7.1^* for intermediate levels, *UAS-AOX^8.1^* and *UAS-AOX^7.1^* [18] for weak 1 and 2 levels, and *UAS-mutAOX^2nd^;UAS-mutAOX^3rd^* for the catalytically inactive mutant form of AOX [18]. Uninduced controls are progeny of the same *UAS* lines and *w^1118^*. (**B**) Immunoblot showing levels in whole body samples of a mitochondrial protein marker, ATP5A, and AOX when its expression is ubiquitously induced at strong (S), intermediate (I) or weak (W1, W2) levels on the indicated diet, using the same lines as in (**A**). M, mutant AOX line, which shows expression lower than expected and partial degradation of the mutant protein. The 50 and 37 kDa bands of the Bio-Rad Precision Plus Protein Standards marker are shown next to the blots. (**C**) Pupal viability of control flies and flies with tissue-specific induction or not (unind hemiz controls) of the strong expression of AOX in muscles, neurons, or fat body, when cultured at the indicated diet. bkg, genetic background; homoz, homozygous; hemiz, hemizygous; unind, uninduced. The homoz driver and unind homoz controls represent, respectively, the parental *GAL4* driver and *UAS* lines. The hemiz driver and unind hemiz controls represent, respectively, flies with a single copy of the transgenic *GAL4* and *UAS* constructs, i.e., progeny of the cross between the homozygous lines with *w^1118^*. Tissue-specific induction is accomplished by crossing homozygous *GAL4* with *UAS* lines, resulting in flies with a single hemizygous copy of each construct. GFP, *UAS-GFP^Stinger^*; strong AOX, *UAS-AOX^F6^* [12]. The data in (**A**,**C**) represent the average of two or three experiments; error bars represent standard deviations. In (**A**), a and b represent significantly different statistical classes (multifactorial ANOVA, followed by the Tukey post hoc test, *p* < 0.05). In (**C**), a multifactorial ANOVA is applied, but no statistical differences are found. Original images of (**B**) can be found in Supplementary Materials.

The *UAS-AOX*, *UAS-GFP^Stinger^* and *UAS-empty* lines were crossed to either a *GAL4* driver line to induce transgene expression, or to the recipient line *w^1118^* (generating uninduced hemizygous controls—these controls were also used in the *Measurements of body mass and composition*, *Immunoblotting* and *Confocal microscopy imaging* experiments), as indicated in the figure legends. Based on immunoblot analyses (Figure 1B) and previously published work [18], the crosses between *UAS-AOX^F6^* and the *GAL4* drivers generated flies with high expression levels of AOX, whereas the same crosses using *UAS-AOX^8.1^* or *UAS-AOX^7.1^* produced low levels of AOX. The double transgenic lines *UAS-AOX^8.1^;UAS-AOX^7.1^* and *UAS-mutAOX^2nd^;UAS-mutAOX^3rd^*, upon crossing with *GAL4* drivers, generated flies with intermediate expression levels of AOX. In total, 10–15 female virgins and 5–7 males were pre-mated on the SD diet during 24–48 h at 25 °C with a 12/12 h light/dark photoperiod cycle, flipped into vials containing SD or LN diets, and allowed to lay eggs until the limiting rate of 60–80 eggs per vial was reached. The eggs were allowed to develop in the same vials, from which the numbers of pupae and eclosing adults were recorded. Pupal viability was calculated as the ratio between eclosed adults and the total number of pupae, averaged among 2–3 biological replicates, each carrying 5–8 vials (technical replicates). Larval mobility was calculated as the average distance crawled in 1 min per third-instar (L3) larva, as described in Garcia et al. [35].

### 2.3. Measurements of Body Mass and Composition

For larval body mass measurements, batches of ten randomly selected, wandering L3 larvae (because the LN diet delays development [24,25], we used larvae of 100–115 h post-egg laying in SD diet and 125–140 h in LN diet) were collected, briefly rinsed in deionized water, carefully dried on Kimwipes^®^ sheets (Kimberly-Clark Inc., Irving, TX, USA), placed in a pre-weighed 1.5 mL microcentrifuge tube, and weighed using a precision balance (ATX224, Shimadzu Scientific Instruments, Canby, OR, USA) to obtain the wet mass. The tubes were then frozen at −20 °C for up to 14 days and placed in a dry bath at 65 °C with the lids open until the larval weight dropped to constant values (~2 h), which were recorded as the dry mass. Next, the samples were incubated with a 1 mL ethyl ether for 24 h at room temperature, and the procedure was further repeated three times, after which the samples were dried as described above, to obtain the lean mass. Individual larval masses were calculated by dividing the total weight per tube by 10, and the final values were obtained by averaging the individual larval masses of ~40 tubes, obtained in three independent experiments. Measurements of total fat content were obtained by subtracting lean from dry mass.

For estimates of total protein content, batches of 20 randomly selected, wandering L3 larvae were collected, briefly rinsed in deionized water, carefully dried on Kimwipes^®^ sheets, and homogenized on ice by 3–5 strokes of a hand-held homogenizer in a 1 mL isolation buffer (250 mM of sucrose, 5 mM of Tris-HCl, 2 mM of EGTA, pH 7.5). The total protein content was estimated by the Bradford method. Individual larval protein content was calculated by dividing the total protein per tube by 20, and the final values were obtained by averaging data from 4 to 6 independent experiments.

### 2.4. Immunoblotting

Mitochondrial protein extracts were prepared from 20 randomly selected, whole adult males (1–3 days after eclosion). Females were excluded to avoid the inherent variability in mitochondrial content throughout the lifespan [36]. Individuals of the indicated genotype were placed in isolation buffer (250 mM of sucrose, 5 mM of Tris-HCl, 2 mM of EGTA, pH 7.5) on ice and homogenized, followed by centrifugation at 200× *g_max_* for 1 min at 4 °C. The supernatant was removed and centrifuged again at 200× *g_max_* for 3 min at 4 °C. The recovered supernatant was further centrifuged at 9000× *g_max_* for 10 min at 4 °C, and the mitochondrial pellet was resuspended in a 50 μL isolation buffer. The protein quantification was performed by the Bradford method, and the samples were stored at −80 °C until use. Forty μg of mitochondrial protein extract were mixed with Laemmli buffer (2% SDS, 10% glycerol, 5% 2- mercaptoethanol, 0.02% bromophenol blue and 62.5 mM of Tris-HCl, pH 6.8), denatured at 95 °C for 3 min, and resolved on 12% SDS-polyacrylamide gels for ~3.5 h at 80 V, alongside the Bio-Rad Precision Plus Protein Standards marker. The proteins were transferred at 550 mA for 7 min to nitrocellulose membranes using the semi-dry transfer system MSD10 (Major Science Inc., Saratoga, CA, USA) at room temperature. The membranes were blocked using 5% nonfat milk/PBST solution (8 mM of Na_2_HPO_4_, 2 mM of KH_2_PO_4_, 150 mM of NaCl, 30 mM of KCl, 0.05% Tween 20, 5% dried nonfat milk, pH 7.4) for at least 2 h, and incubated with a 1% nonfat milk/PBST solution containing the primary antibodies anti-AOX (rabbit polyclonal, 1:20,000) [12] and anti-ATP5A (mouse monoclonal, Abcam Inc., Cambrigde, MA, USA, 1:10,000) for 3 h at room temperature under constant shaking. After three 40 min washes in PBST, the membranes were incubated with a 1% nonfat milk/PBST solution containing the secondary HRP-conjugated goat anti-rabbit (1:10,000, Bio-Rad, Kidlington, UK) and anti-mouse (1:10,000, Bio-Rad, Kidlington, UK) antibodies overnight at 4 °C, and washed as described. Chemiluminescence signals were detected on a ChemiDoc Imaging System (Bio-Rad, Richmond, CA, USA), after the membranes were incubated with the luminol substrate Immun-Star HRP (Bio-Rad, Kidlington, UK).

### 2.5. Metabolite Extraction and Mass Spectrometry Analyses

Ten randomly selected, wandering L3 larvae (100–115 h post-egg laying in SD diet; 125–140 h in LN diet), progeny of *UAS-AOX^F6^* and *daGAL4* (AOX-expressing) and of *w^1118^* and *daGAL4* (control), per biological replicate (5 in total) were collected, briefly rinsed in ultrapure water, and homogenized in 1 mL of cold (−80 °C) 80% methanol on dry ice using a probe homogenizer. Samples were clarified by centrifugation for 5 min at 16,000× *g_max_* at 4 °C, and the supernatants were transferred to new microcentrifuge tubes. The remaining pellets were resuspended in RIPA lysis buffer (10 mM of Tris-HCl, 150 mM of NaCl, 1% Triton X-100, 0.1% deoxycholate, 0.1% SDS, pH 7.5), and the total protein content was determined by the BCA method. A volume of the supernatant equivalent to 5 µg of protein was transferred to a glass vial, dried using an EZ-2 Elite evaporator (SP, Warminster, PA, USA), and stored at −80 °C until analysis by LC-MS. The samples were analyzed using an UltiMate 3000RSLC HPLC (Thermo Scientific, Waltham, MA, USA) coupled to a Q Exactive mass spectrometer (Thermo Scientific, Waltham, MA, USA). After resuspension in 50% acetonitrile, 10% of each sample was loaded onto a Luna NH2 (3 µm 100 A, 150 mm × 2 mm, Phenomenex, Torrance, CA, USA) column. Separation was achieved with 5 mM of NH_4_OAc, pH 9.9 (mobile phase A) and ACN (mobile phase B) at a flow rate of 200 µL/min and a linear gradient from 15% to 90% A over 18 min. This was followed by an isocratic step at 90% A for 9 min and re-equilibration to the initial 15% A for 7 min. The Q Exactive was run with polarity switching (+3.50 kV/−3.50 kV) in full scan mode with an *m*/*z* range of 65–975.

Metabolites were identified with TraceFinder 4.1 (Thermo Scientific, Waltham, MA, USA) using accurate mass measurements (≤3 ppm) and expected retention times previously established with pure standards. Quantities were determined by peak area integration, and the statistical analysis was performed using the MetaboAnalyst5.0 software [37]. Missing values were replaced by 1/5 of minimum positive values of the corresponding variables. Following the normalization step, a two-way type I analysis of variance (ANOVA) was performed, including the interaction between the AOX and LN factors, and the metabolites significantly altered were determined as *p*-adjusted value < 0.05.

### 2.6. Confocal Microscopy Imaging

The musculature of L3 larvae or adult thorax was dissected in phosphate-buffered saline (PBS) using thin dissection forceps, fixed in 4% paraformaldehyde for 20 min at room temperature, and then transferred to PBS prior to permeabilization. The samples were permeabilized and blocked with PBS containing 1% Triton X-100 and 1% bovine serum albumin (BSA) for 30 min at room temperature, followed by overnight incubation at 4 °C with the primary antibody diluted in PBS containing 0.3% Triton X-100 and 0.5% BSA. The samples were then washed three times (30 min each) with PBS containing 0.1% Triton X-100 and 0.1% BSA at room temperature, followed by overnight incubation at 4 °C with the secondary antibody diluted in PBS containing 0.3% Triton X-100 and 0.5% BSA. Washes were performed as described above, and samples were rinsed with PBS and then Milli-Q water, prior to transferring to slides containing Fluoromount Aqueous Mounting medium (Sigma-Aldrich, St. Louis, MO, USA). Primary antibodies used were rabbit polyclonal anti-AOX (1:10,000) [12] and mouse monoclonal anti-ATP5A (1:1000, Abcam Inc., Cambrigde, MA, USA). Fluorescent secondary antibodies were Alexa 488-conjugated goat antirabbit IgG (1:1000) and Alexa 568-conjugated goat antimouse IgG (1:1000) (Fisher Scientific, Pittsburgh, PA, USA). Actin and nuclei were stained using phalloidin-tetramethylrhodamine B isothiocyanate (TRITC) (1 μg/mL, Sigma-Aldrich, St. Louis, MO, USA) and 4′,6-diamidino-2-phenylindole (1 μg/mL, Molecular Probes, Eugene, OR, USA). Samples were imaged using a Leica SP8 confocal microscope and the LAS X software, v. 1.4.7. Images were analyzed with ImageJ software, v. 1.54j (National Institutes of Health, Bethesda, MD, USA).

### 2.7. Statistical Analyses

Statistical analyses for the mass spectrometry data were performed as described above. The remaining analyses were performed using the Jamovi, v. 2.5, (https://www.jamovi.org) or GraphPad Prism, v. 8.0.1, software, as described in the figure legends.

## 3. Results

### 3.1. Low-Nutrient Diet and AOX Expression Cause Changes in Amino Acid Metabolism and a Severe Drop in Larval Biomass

In a previously published study [24], we showed that the LN diet-dependent pupal lethality was achieved by high levels of ubiquitously driven AOX expression. Here, we analyzed additional *UAS-AOX* lines that, when crossed with the *daGAL4* driver line, provided flies with varying levels of ubiquitous wild-type AOX expression, assigned as “weak”, “intermediate” and “strong”, or with the expression of a catalytically inactive mutant of AOX [18]. As uninduced controls, we used the progeny of the *UAS-AOX* lines and the background line *w^1118^*. We confirmed that, in the LN diet, pupal lethality only occurs with a strong expression of wild-type AOX (Figure 1A), suggesting that the phenotype is dependent on a threshold effect. We also verified that the LN diet does not alter AOX protein levels (Figure 1B). From this point on, unless otherwise stated, we continued to analyze only the flies with strong AOX expression (*daGAL4*-driven progeny of the line *UAS-AOX^F6^* [12]), referring to them simply as AOX-expressing flies.

**Figure 2:**
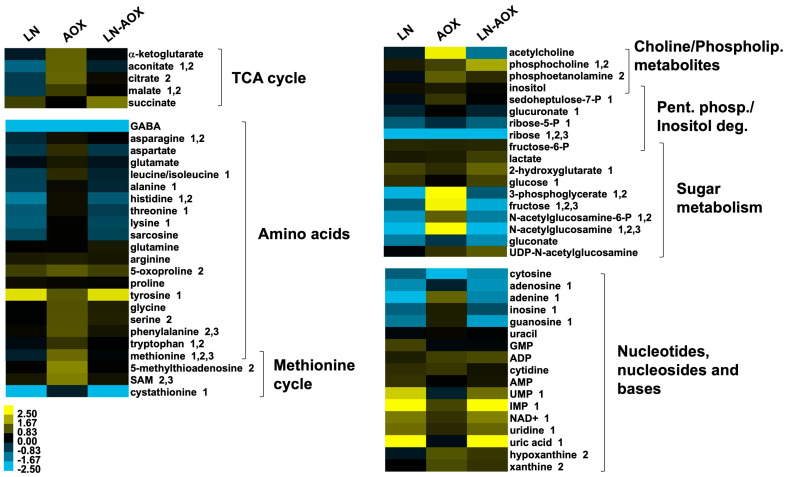
Larval amino acid and TCA cycle intermediate levels are differentially affected by diet and AOX expression. Log_2_ (fold change) values of the indicated metabolites in control larvae cultured on the LN diet, AOX-expressing larvae cultured in the SD diet, and AOX-expressing larvae culture in the LN diet, each in a pairwise comparison with control larvae cultured in the SD diet. 1–3, respectively, denote metabolites significantly altered by the LN diet, AOX expression and the LN-AOX interaction (two-way ANOVA, *p* < 0.05). The data represent the average of six biological replicas. See Supplemental Table S1 for the full list of metabolites. TCA, tricarboxylic acid; Phospholip., phospholipid; Pent. Phos., pentose phosphate pathway; Inositol deg., inositol degradation pathway. Control larvae, progeny of *w^1118^* and *daGAL4*; AOX-expressing larvae, progeny of *UAS-AOX^F6^* and *daGAL4*.

We then applied a metabolomic approach to investigate the whole-body larval metabolism of AOX-expressing (progeny of *UAS-AOX^F6^* and *daGAL4*) and control larvae (progeny of *w^1118^* and *daGAL4*) cultured on SD and LN diets, and to enable us to develop a mechanistic hypothesis. A multifactorial analysis of samples from wandering L3 larvae revealed that the LN diet causes significant alterations in the levels of 55% of the 74 unique metabolites identified (Supplemental Table S1). As expected, the LN diet promoted decreased levels of most intermediates of the TCA cycle and the pentose phosphate pathway, most amino acids, and a general rearrangement in the levels of nitrogenous bases, nucleosides and nucleotides, including an increase in markers of energy depletion such as nucleotide monophosphates and NAD+ (Figure 2). Globally, these changes are consistent with a high energy demand for the biosynthesis and storage of new biomolecules (characteristic of the proliferative nature of larval tissues) combined with a low supply of high-energy nutrients from the diet.

**Figure 3:**
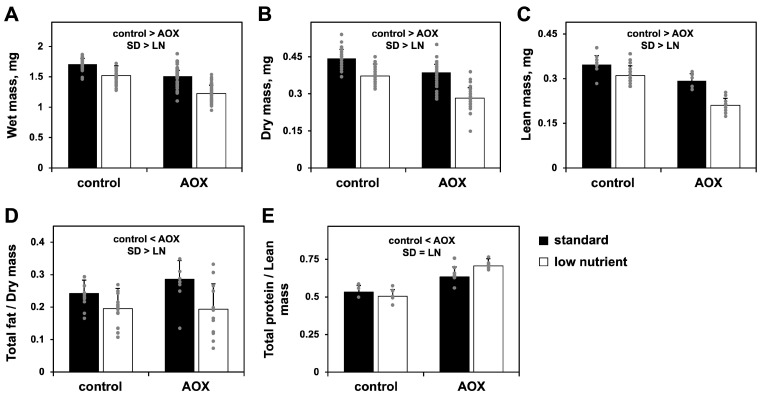
The combination of AOX expression and LN diet promotes a dramatic decrease in larval body mass: wet (**A**), dry (**B**) and lean (**C**) masses and total fat (**D**) and protein (**E**) of L3 larvae of the indicated genotypes, cultured on diets as shown. The data represent averages of three biological replicas, each performed with 8–20 technical replicas, in (**A**,**B**); and, of two biological replicas, each performed with 3–10 technical replicas, in (**C**–**E**). Error bars represent standard deviations. Control, progeny of *UAS-AOX^F6^* and *w^1118^*; AOX, progeny of *UAS-AOX^F6^* and *daGAL4*. “control > AOX” and “control < AOX” indicate significant differences between control and AOX-expressing flies; “SD > LN”, between diets; “=” symbol, no significant difference within a factor (two-way ANOVA, *p* < 0.05). No significant interactions between factors are found.

AOX expression, on the other hand, caused alterations in 28% of the identified metabolites (Supplemental Table S1) but with the opposite tendency of what is observed for the LN diet (Figure 2). In the LN-AOX interaction, the diet appears to prevent most of the metabolomic changes induced by AOX. The general increase in the levels of most amino acids in AOX-expressing larvae cultured in the SD diet is of particular interest as, in addition to their need in protein synthesis and their interconnections with sugar, nucleotide and lipid metabolites needed during tissue proliferation [38,39], they play important roles in cell signaling [40]. We thus checked if these contrasting alterations in amino acid and other metabolite levels would correlate with larval biomass accumulation. Although developing normally, AOX larvae cultured on the SD diet had a body mass similar to that of control larvae (progeny of *UAS-AOX^F6^* and *w^1118^*) on the LN diet, decreased by ~15% compared to the same controls on the SD diet. The effects of the two conditions together were additive, leading to a ~40% decreased body mass (Figure 3A–C) in the LN-AOX larvae, accompanied by a decrease in larval and pupal size (although the data for pupal size were not statistically significant; Supplemental Figure S1A,B,D). We also quantitated the total fat and protein content in our larvae and observed that, whereas fat is lowered by the LN diet irrespective of genotype, AOX expression increases fat and protein levels, but the increase in fat is SD diet-dependent (Figure 3D–E). These results suggest complex metabolic rerouting induced by AOX expression, with lethal consequences in LN conditions.

### 3.2. Derangement of Myofibril Structure Is a Hallmark of AOX-Expressing Larvae Cultured on Low-Nutrient Diet

The increased protein content but generally low amino acid levels in LN-AOX larvae prompted us to investigate organ development. We began with the L3 larval musculature as, in addition to allowing larval movement through peristalsis, the organ serves as an important source of stored protein for pupal development [41]. We first confirmed that AOX driven by *daGAL4* is present in this tissue and that it colocalizes with mitochondria (Supplemental Figure S2). The number of muscle mitochondria, which are almost all round-shaped and dispersed throughout the fibers, does not change significantly, although the expression of AOX appears to lead to larger mitochondria, especially in the LN diet (Supplemental Figure S2).

**Figure 4:**
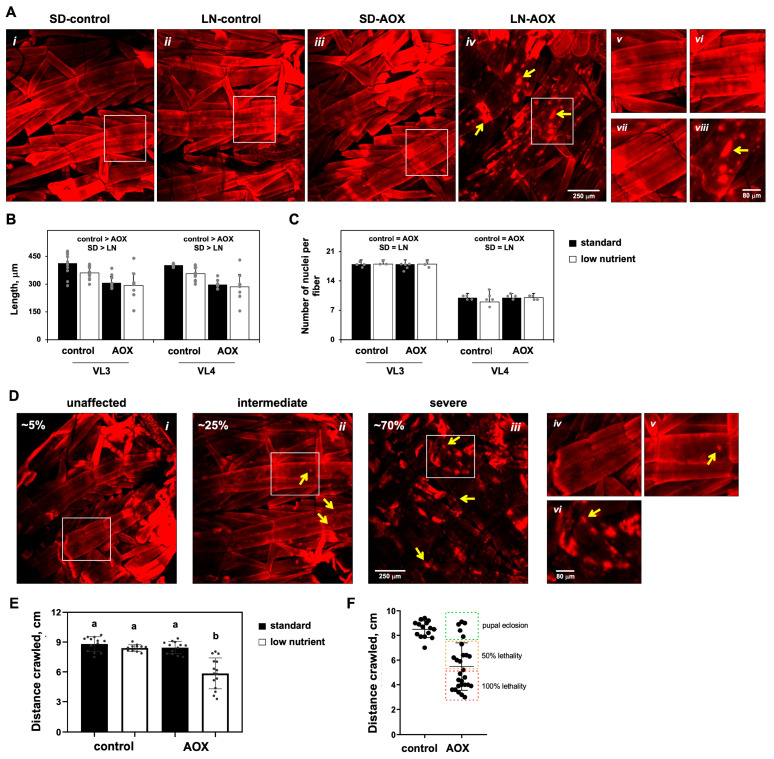
LN diet and AOX expression leads to derangement of the larval musculature, larval locomotor problems and pupal lethality: (**A**) Representative confocal microscopy images of the L3 larval musculature stained with phalloidin-TRITC to detect actin. Panels *v*–*viii*, higher magnification of the quadrants (white rectangles) indicated in *i*–*iv*, respectively. Quantitation of length ((**B**), average of 12 experiments) and number of nuclei ((**C**) average of 3–6 experiments) of the larval longitudinal fibers VL3 and 4 of the indicated flies cultured on the indicated diet. “control > AOX” and “SD > LN” indicate significant differences between control and AOX-expressing flies, and between diets, respectively; “=” symbol, no significant difference within a factor (two-way ANOVA, *p* < 0.05). No significant interactions between factors are found. (**D**), Representative confocal microscopy images of the L3 larval musculature of AOX-expressing individuals cultured on the LN diet, stained with phalloidin-TRITC to detect actin. Percentages indicate the proportion of individuals with the indicated phenotypic classes. Panels *iv*–*vi*, higher magnification of the quadrants (white rectangles) indicated in *i*–*iii*, respectively. The *yellow arrows* in (**A**,**D**) are examples of actin molecule agglomerations (see text for more details). (**E**) Motility of larvae of the indicated genotype cultured on the indicated diet (average of 11–15 experiments). (**F**) Combined analyses of larval motility and pupal lethality/eclosion of groups of larvae of the indicated genotype, cultured on the LN diet (average of 15–27 experiments). The error bars represent standard deviations in all graphs. SD and LN, standard and low-nutrient diets; control, progeny of *UAS-AOX^F6^* and *w^1118^*; AOX, progeny of *UAS-AOX^F6^* and *daGAL4*. a,b represents different statistical classes according to a two-way ANOVA, followed by the Tukey post hoc test.

Actin staining revealed that general muscle morphology is unaltered by the diet or AOX expression (Figure 4A), although these two factors individually shorten the musculature by 10–20%, as judged by the length of the major longitudinal fibers VL3 and 4 (Figure 4B). The majority (~70%) of LN-AOX larvae, on the other hand, showed severe alterations in the structure and localization of actin, with no changes in VL3 and 4 length (Figure 4A,B). Actin molecules in LN-AOX larval muscle appear to agglomerate in “blobs” randomly located inside the muscle fibers (Figure 4A, *yellow arrows*). This phenotype was heterogenous: approximately 5% of the LN-AOX individuals analyzed presented muscle morphology similarly to the controls, and about 25% had an intermediate phenotype, in which the actin fibers still appear intact, but with detectable actin “blobs” (Figure 4D). We also quantitated the number of nuclei in each VL3 and 4 fibers, as changes in nuclei number could indicate problems in embryonic myoblast fusion [42] and might explain the shorter fiber phenotype. No significant changes were detected (Figure 4C), which is consistent with the idea that the muscle phenotype seen in Figure 4A develops post-embryonically, when the AOX-expressing individuals start utilizing the LN diet instead of the egg nutrient storage deposited by the mother, failing to obtain key metabolite(s) for normal growth and development.

To test if such a strong muscle phenotype would have an impact in actin function and, thus, in muscle contraction, we measured the motility of L3 larvae and observed an almost 40% decrease in the crawling ability of LN-AOX individuals (Figure 4E). Most importantly, we noticed a high variability in these larvae’s motility, with some individuals crawling as much distance as the controls, and others performing very poorly (decreased motility up to 65%, compared to the average of the control progeny of *UAS-AOX^F6^* and *w^1118^*). We then separated the LN-AOX larvae into three categories based on their motility skills and observed a positive correlation among crawling abilities, deranged muscle morphology phenotype, and lethality at the pupal stage (Figure 4F). For example, most larvae with normal locomotion had normal muscle morphology and eclosion rates, whereas individuals with the intermediate locomotion phenotype had also intermediate muscle morphology phenotype and ~50% pupal lethality (Figure 4F). We also analyzed the morphology of the thoracic dorsal longitudinal muscle of LN-AOX eclosed adults (the escapers) and noticed no apparent changes and no differences in sarcomere length when compared to AOX-expressing adults cultured on the SD diet and to AOX nonexpressors (Supplemental Figure S3). These data clearly indicate that larval muscle development and locomotion in LN-AOX individuals is a strong predictor of adult eclosion rates and that the LN-AOX individuals that reach the adult stage carry no muscle morphological alterations. The data also support heterogeneity in the development of the muscle phenotype.

### 3.3. Rescue of the Phenotypes of AOX-Expressing Flies Cultured on LN Diet by Amino Acid Supplementation

To further test if the lethality of the AOX-expressing flies in the LN diet was due to muscle-specific effects, we drove expression of AOX in three larval tissues of major importance for whole-animal energy metabolism. AOX expression in the larval musculature, nervous system, or fat body, in combination with the LN diet, had no effect on pupal viability (Figure 1C). The LN diet and AOX expression solely in the larval musculature also did not cause myofibril derangement. The lethal phenotype of AOX driven by *daGAL4* must therefore reflect a systemic effect initiated across one or more tissues and impacting muscle function.

**Figure 5:**
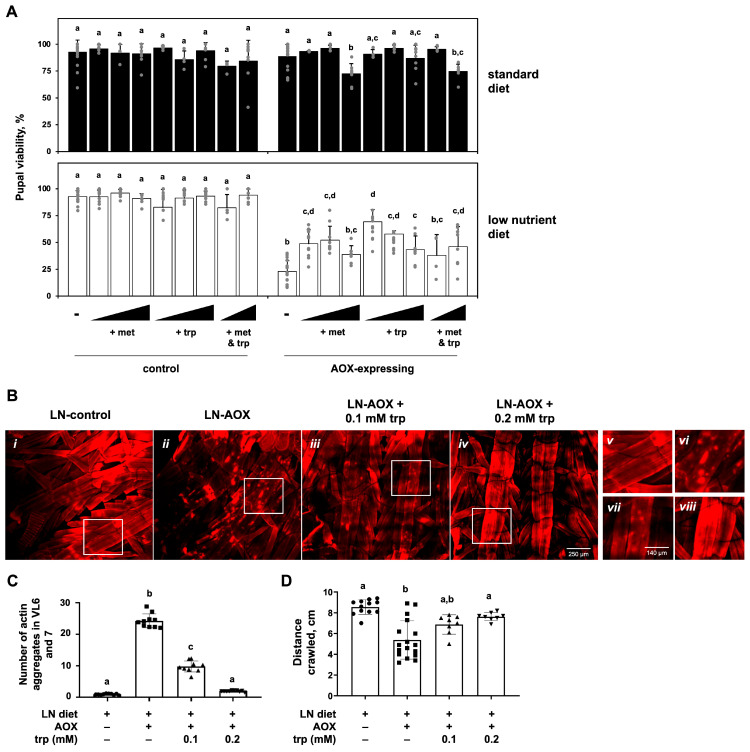
Rescue of pupal lethality, larval motility and muscle aberrations of AOX-expressing flies cultured on the LN diet supplemented with methionine/tryptophan: (**A**) Pupal viability (adult eclosion percentage, average of 4–20 experiments) of flies of the indicated genotypes, cultured on the diet and supplements as shown. The final concentrations of added amino acids are as follows: methionine (met—0.18, 0.35 and 0.7 mM; tryptophan (trp)—0.1, 0.2 and 0.4 mM). These concentrations and those used in Supplemental Figure S4 are selected based on a previously published study [43]. (**B**) Representative confocal fluorescent microscopy images of L3 larvae muscle fibers of the indicated genotypes (panels *i*–*iv*), showing a decrease in actin agglomeration foci when the LN diet is supplemented with the indicated concentration of tryptophan. Panels *v*–*viii*, higher magnification of the quadrants (white rectangles) indicated in *i*–*iv*, respectively. (**C**) Quantitation of the data shown in B plus two other biological replicates, each with 3–4 technical replicas. (**D**) Larval motility of flies of the indicated genotype cultured on the LN diet with the indicated concentration of tryptophan (average of 8–17 experiments). Error bars represent standard deviations in all graphs. a–d indicate different statistical classes, according to multifactorial ANOVAs, followed by the Tukey post hoc test. Control, progeny of *UAS-AOX^F6^* and *w^1118^*; AOX-expressing, progeny of *UAS-AOX^F6^* and *daGAL4*.

Based on the metabolomic data shown on Figure 2, we decided to test the supplementation of the SD and LN diets with specific amino acids and check for rescue of the phenotypes associated with the ubiquitous expression of AOX. The addition of the essential amino acids methionine and tryptophan, individually and in combination, caused a significant, albeit partial, rescue of pupal lethality (Figure 5A) and body size (Supplemental Figure S1B–E) of AOX flies cultured on the LN diet. Restoration of pupal viability was most apparent with low or intermediate doses of the amino acids and were not additive, implying that a correct balance of these molecules is required for development and that they are contributing to the same pathway. In contrast, supplementing the LN diet with the non-essential amino acids proline and/or glutamate, despite their strong bioenergetic potential, failed to rescue the AOX-induced lethality (Supplemental Figure S4), suggesting that energy availability is not likely a limiting factor. Tryptophan supplementation also rescued the muscle morphology and locomotion of AOX-expressing larvae exposed to the LN diet (Figure 5B–D). Altogether, our data indicate that the development of AOX-expressing flies depends on specific dietary essential amino acids to maintain healthy larval muscle function.

## 4. Discussion

An OXPHOS bypass using AOX has been proposed as a potential strategy for treating human mitochondrial and related diseases [11,20]. To validate its safety, assessing the effects of AOX expression across diverse animal models and experimental conditions is crucial. We confirm here that the development of flies with a strong ubiquitous expression of AOX is significantly impacted when cultured on an LN diet. LN-AOX larvae exhibit defects in biomass accumulation, musculature organization, and motility, leading to high levels of mortality at the pupal stage. In a sense, the lethality phenotype is similar to those shown for OXPHOS defects in flies [5,44,45,46], arguing that the LN-AOX combination may be treated as a mitochondrial disease-like condition.

We previously attempted to provide extra dietary protein by increasing the concentration of yeast extract up to 10% (*w*/*w*) into the LN diet and observed no improvement in pupal viability upon AOX expression [25]. Although rich in proteins, yeast extract generally contains proportionally low levels of methionine, tryptophan, and cysteine [47]. The addition in the LN diet of the water-soluble fraction of molasses, which is rich in methionine, tryptophan, arginine, valine, glutamine, cysteine and aspartate, rescued the lethal phenotype induced by the LN-AOX interaction [25]. Here, we show that larval amino acid levels are generally decreased by the LN diet while increased upon AOX expression. Methionine stands out as the only amino acid whose levels are altered in LN, AOX, and the LN-AOX interaction. In contrast, tryptophan levels are normalized in LN-AOX larvae, and proline and glutamate remain unaffected by any of these conditions. Because of these differences and our previous coarse complementation assays [22], we selected these four amino acids for additional complementation studies. We partially rescued the LN-AOX defects by LN supplementation with methionine or tryptophan but not proline or glutamate, indicating a specific importance of the former metabolites in conditions of low nutrient availability combined with AOX expression.

In addition to methionine, several metabolites of the methionine cycle were also elevated in AOX-expressing larvae (Figure 2). The methionine cycle is important for the cellular production of the universal methyl donor *S*-adenosylmethionine (SAM), used for epigenetic regulation of the nuclear genome [48,49]. It is also interconnected with the metabolism of several other amino acids and nucleotides needed during tissue proliferation [38,39]. The amino acids serine, glycine and tryptophan, among others, can fuel the folate cycle, which in turn provides methyl groups via 5-methyl-tetrahydrofolate (5-methyl-THF) to the methionine cycle; together, the two cycles are referred to as one-carbon metabolism [50,51,52].

In addition to its role as building blocks of proteins, tryptophan plays a key function in signaling. In particular, it is the precursor of serotonin (5-hydroxytryptamine), involved in development through the modulation of cyclins and cyclin-dependent kinases, neuronal growth and differentiation, and the regulation of circadian genes. Its disruption leads to abnormal locomotion and feeding behavior, mimicking neurodevelopmental disorders [53,54]. Through the kynurenine pathway, tryptophan is also an important precursor for NAD+ synthesis [40,55]; its depletion is therefore able to influence intracellular signaling, cellular health and homeostasis, and responses to stress, primarily through the metabolism regulator SIRT1 [56]. Although dysfunction of the kynurenine pathway in *Drosophila* is associated with defects in the synthesis of the brown pigment of the eyes [57,58] and with alterations of mitochondrial dynamics and turnover [59], we did not observe a change in eye color due to diet nor to AOX expression. Future tests based on mitochondrial imaging and the analysis of pigment variation in eye-color mutants of *Drosophila* in the context of AOX expression may help reveal which tryptophan metabolic pathway(s) is(are) causal in the LN-AOX lethality.

We previously found that additions of extra sugar to the LN diet did not improve the survival of LN-AOX flies [25], like proline and/or glutamate supplementation (Supplemental Figure S4). These results are consistent with the idea that yeast extract alone has an imbalanced amino acid content that fails to restore the proper development of AOX-expressing flies in LN diet. Since sugar, proline and glutamate oxidation would provide reducing power for ATP synthesis via OXPHOS, we propose that energy production per se is not likely a limiting factor for LN-AOX larval metabolism. Although all our fly lines are in the *w^1118^* background, which harbors low levels of a tryptophan transporter [60], our numerous control crosses (Figure 1) produced flies with varying expression levels of the transporter-coding gene, *white* (used as marker for the original transgenesis events that created the lines), with no apparent effect on viability on LN diet, confirming that the lethality is a specific consequence of AOX expression. Complete rescue of the AOX-driven pupal lethality, as seen with the addition of molasses to the LN diet [25], might require a combination of proper levels of methionine or tryptophan, plus other nutrients/molecules, which are yet to be identified. This is further supported by the fact that tryptophan supplementation appears to promote the almost full rescue of myofibril organization and larval motility, although it fails to completely restore pupal viability rates. Elucidating the effects of amino acids on the diverse AOX-induced phenotypes may not be straightforward, as, even on an SD diet, the highest dose of methionine supplementation tested (Figure 5A) caused a significant degree of lethality in AOX-expressing flies, indicating that an optimal dose is necessary, above and below which viability decreases. Testing amino acid metabolism in the context of the larval AOX expression is thus an import future avenue of research.

We also previously observed that triglycerides were significantly lower in larvae cultured on LN, but AOX expression had no effect on the levels of these storage molecules [25]. Because, in *Drosophila*, triglyceride levels increase as the larvae grow and continue increasing during metamorphosis at the expense of membrane lipids [61], we quantitated the total fat content in our larvae by ether extraction to derive an estimate for combined triglycerides, membrane lipids, and other lipids. The LN diet lowered total larval fat, which is consistent with the drop in triglycerides previously seen [25]. In contrast, AOX-expressing larvae showed increased fat content, which is apparent only in the SD diet (Figure 3D). We also estimated the total content of larval protein, as this is another class of stored nutrients important for pupal development [41]. AOX expression was associated with a 25–40% increase in total protein levels, irrespective of diet (Figure 3E).

Taken together, our data indicate that AOX-expressing larvae accumulate more fat and protein for a given amount of larval mass, when cultured on SD diet. This may indicate a successful compensatory adaptation to decreased mitochondrial energy efficiency, achieved through increased nutrient storage in the larvae, allowing the flies to traverse metamorphosis and reach the adult stage, despite the possible energy dissipation caused by AOX activity. This might even be related to the effects of AOX expression on signaling mechanisms important for development, as evidenced through its interference with the JNK pathway [22], a phenomenon that is still to be fully understood. In the LN diet, where yeast extract is the sole nutrient source, the increase in fat accumulation appears to be suppressed by AOX expression. We speculate that the AOX-associated changes are likely due to altered nutrient absorption, geared to enable larvae to accumulate sufficient nutrients for the completion of development. This is sufficient for normal development of the flies in the SD diet, but, in the LN diet, the uncoupling effects of AOX cannot be compensated, leading to metabolic imbalance in larvae and wasting in pupae. It is possible that the actin agglomerations observed in the musculature of LN-AOX larvae are products of early apoptotic events, which occur when larval fat storage levels are critically low, in an attempt to provide nutrients for essential fly functions. However, how these and other alterations relate to fly survival remain to be addressed in future studies.

Because the developmental arrest described here is likely dependent on a systemic impact caused by the strong ubiquitous expression of AOX in combination with an LN diet, the metabolic and signaling changes induced by AOX possibly depend on the functions of particular tissues or organs. AOX expression might influence general larval growth by acting locally in each tissue/organ, but the problems seen on the LN diet occur with the integration of these organs’ functions. By applying whole-larva metabolomics, we may have missed important tissue-specific signals and metabolic changes promoted by AOX, but, in the future, we may be able to identify important signaling metabolite(s) with a more systemic role and an expected impact beyond metabolism and protein synthesis. For example, tryptophan and methionine play a key role in antioxidant response, DNA methylation, neurogenesis, cell proliferation, larval behavior and more. Therefore, future studies should focus on profiling systemic as well as tissue-specific metabolic and genetic disturbances caused by AOX in the growing *Drosophila* larva and how they are integrated to promote growth and biomass accumulation (or the lack thereof in an LN diet).

## 5. Conclusions

Our study demonstrates that the combination of an LN diet and AOX expression significantly impacts *Drosophila* larval metabolism, biomass accumulation, and muscle development. AOX expression induces broad metabolic alterations, particularly in amino acid levels, which are counteracted by the metabolic constraints of the LN diet. The combined effects lead to severe reductions in larval size, pupal lethality, and structural abnormalities in larval muscles, ultimately impairing locomotion and viability. Notably, supplementation with the essential amino acids methionine and/or tryptophan partially rescues these phenotypes, suggesting their key role in sustaining proper larval development under AOX expression. Our findings indicate that the AOX-induced metabolic rerouting affects systemic nutrient utilization and muscle function, emphasizing the importance of amino acid balance in mitochondrial metabolism. Given that AOX is being explored as a therapeutic strategy for mitochondrial diseases, understanding its systemic effects under different dietary conditions is crucial. Future studies should focus on tissue-specific metabolic interactions and their influence on organismal growth. Additionally, investigating the roles of AOX in signaling pathways may provide insights into its broader physiological consequences and potential risks when considered for therapeutic applications.

## Data Availability

Raw metabolomic data are deposited in the NIH Common Fund’s National Metabolomics Data Repository (Project ID 3056). Processed data can be found in the Supplemental Table S1. Raw data of the analyses presented in Figure 1, Figure 3, Figure 4 and Figure 5 and the Supplemental Figures can be obtained from the corresponding authors upon request.

## Supplementary Materials

The following supporting information can be downloaded at https://www.mdpi.com/article/10.3390/biom15040570/s1. Figure S1: Changes in larval and pupal sizes caused by AOX expression and dietary interventions. Figure S2: Mitochondrial localization of AOX does not change due to diet. Figure S3: Unchanged muscle morphology of AOX-expressing adults cultured on low-nutrient diet (the escapers). Figure S4: Pupal viability of AOX-expressing flies cultured on LN diet supplemented with proline and glutamate. Table S1: Metabolites identified in AOX-expressing and control larvae cultured on standard (SD) or low-nutrient (LN) diet. Original images of Figure 1B can be found in supplementary materials.
