# Supplementary material for: Impairment of Muscle Function Causes Pupal Lethality in Flies Expressing the Mitochondrial Alternative Oxidase
Source: Biomolecules. 2025 Apr 11;15(4):570. doi: 10.3390/biom15040570 (PMC12024792; doi:10.3390/biom15040570)
Supplement: Supplementary file 1 [file biomolecules-15-00570-s001.zip › Supplemental Figures.pdf]

Supplemental Material

Impairment of muscle function causes pupal lethality in flies expressing the mitochondrial alternative oxidase

Carlos A. Couto-Lima, Sina Saari, Geovana S. Garcia, Gabriel H. Rocha, Johanna ten Hoeve, Eric Dufour & Marcos T. Oliveira

Supplemental Figure Legends

Figure S1. Changes in larval and pupal sizes caused by AOX expression and dietary interventions. A, quantitation of larval area of the indicated flies cultured on the indicated diet. The values indicate averages of 15 individual measurements, and the error bars represent standard deviations. B-C, light microscopy images of representative pupae: SD-control, control flies in standard diet; LN-control, control flies in low nutrient diet; SD-AOX, flies with strong expression of AOX in standard diet; LN-AOX, flies with strong expression of AOX in low nutrient diet; LN+met-AOX, flies with strong expression of AOX in low nutrient diet supplemented with 0.7 mM L-methionine; LN+trp-AOX, flies with strong expression of AOX in low nutrient diet supplemented with 0.4 mM L-tryptophan; LN+met+trp-AOX, flies with strong expression of AOX in low nutrient diet supplemented with both amino acids. D-E, quantitation of pupal length and width of the indicated flies cultured on the indicated diet. The values indicate averages of at least 7-10 individual measurements, and the error bars represent standard deviations. + Met, addition of 0.7 mM L-methionine; + Trp, addition of 0.4 mM tryptophan; + Met & Trp, addition of both amino acids; control, progeny of *UAS-AOX*^{F6} and *w*^{1118}; AOX, progeny of *UAS-AOX*^{F6} and *daGAL4*. a, b and c represent significantly different statistical classes, according to a two-way ANOVA, followed by the Tukey *post-hoc* test. In D-E, a multifactorial ANOVA was applied, but no statistical differences were found.

Figure S2. Mitochondrial localization of AOX does not change due to diet. Upper panels, representative confocal fluorescent microscopy images of L3 larvae muscle fibers of the indicated genotype. Lower panel, quantitation of the number of mitochondria in these fibers (average of 5

experiments). control, progeny of *UAS-AOX*^{F6} and *w*^{1118}; AOX, progeny of *UAS-AOX*^{F6} and *daGAL4*. “control = AOX” and “SD = LN” indicate no differences between control and AOX-expressing flies, and between diets (two-way ANOVA), respectively.

Figure S3. Unchanged muscle morphology of AOX-expressing adults cultured on low nutrient diet (the escapers). A, Representative confocal microscopy images of the dorsal longitudinal fibers of the adult indirect flight muscles stained with TRITC-phalloidin to detect actin. B, Quantitation of sarcomere length of the indicated flies cultured on the indicated diet. The values indicate averages of three biological replicas, each with 6-8 technical replicas, and the error bars represent standard deviations. SD and LN, standard and low nutrient diets; control, progeny of *UAS-AOX*^{F6} and *w*^{1118}; AOX, progeny of *UAS-AOX*^{F6} and *daGAL4*. “control = AOX” and “SD = LN” indicate no differences between control and AOX-expressing flies, and between diets (two-way ANOVA), respectively.

Figure S4. Pupal viability of AOX-expressing flies cultured on LN diet supplemented with proline and glutamate. Adult eclosion percentage (average of 4-10 experiments) of flies of the indicated genotypes, cultured on the diet and supplements as shown. The final concentrations of added amino acids were: proline (pro) and glutamate (glu) – 1.7 and 2.9 mM, respectively. control, progeny of *UAS-AOX*^{F6} and *w*^{1118}; AOX-expressing, progeny of *UAS-AOX*^{F6} and *daGAL4*. a-b represent significantly different statistical classes (multifactorial ANOVA, followed by the Tukey *post-hoc* test, $p < 0.05$).

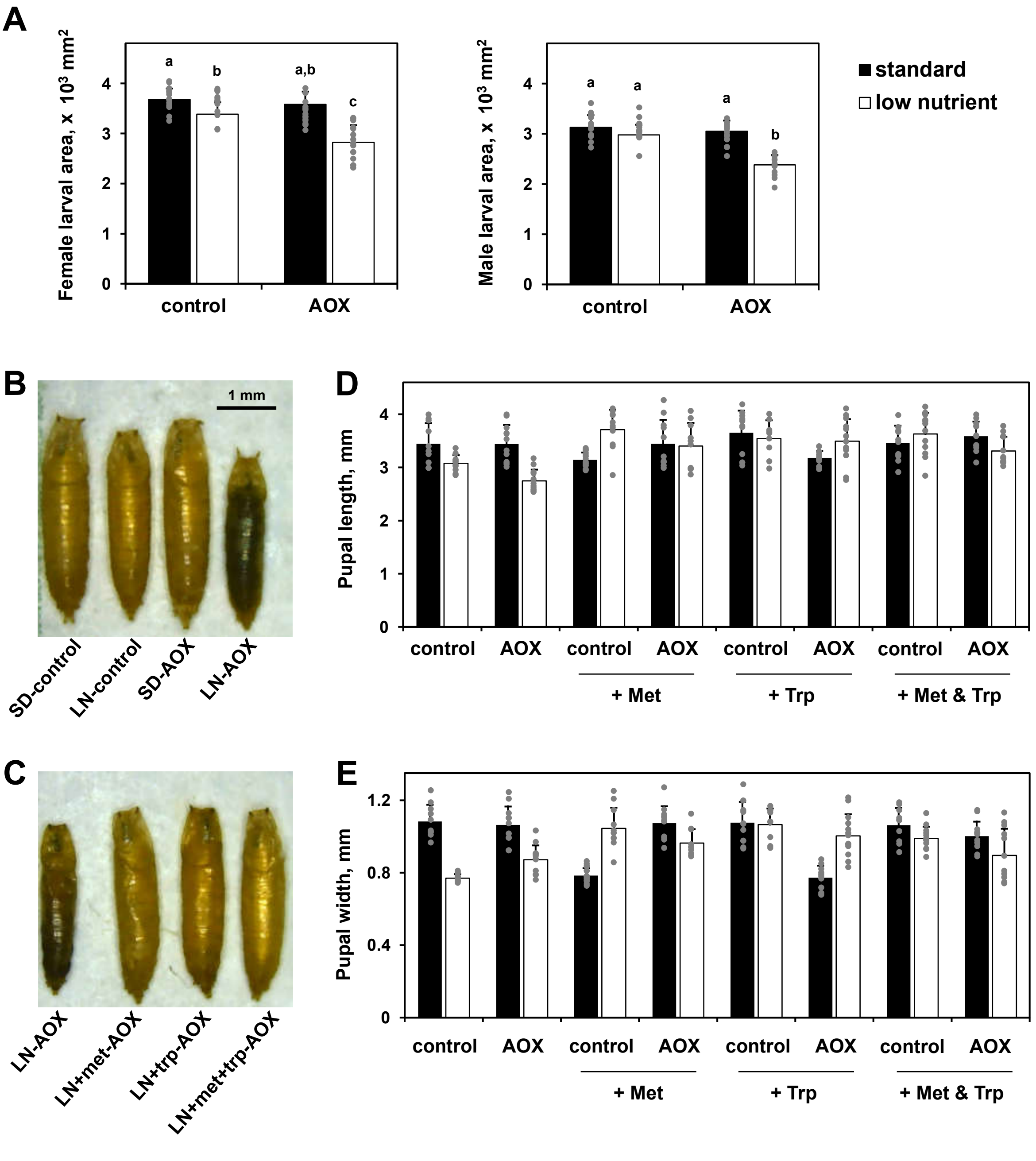
A
Female larval area, x 10³ mm²
Male larval area, x 10³ mm²
a
b
a,b
c
a
a
a
b
control
AOX
standard
low nutrient
B
1 mm
SD-control
LN-control
SD-AOX
LN-AOX
C
LN-AOX
LN+met-AOX
LN+trp-AOX
LN+met+trp-AOX
D
Pupal length, mm
E
Pupal width, mm
control
AOX
+ Met
+ Trp
+ Met & Trp

Figure S1.

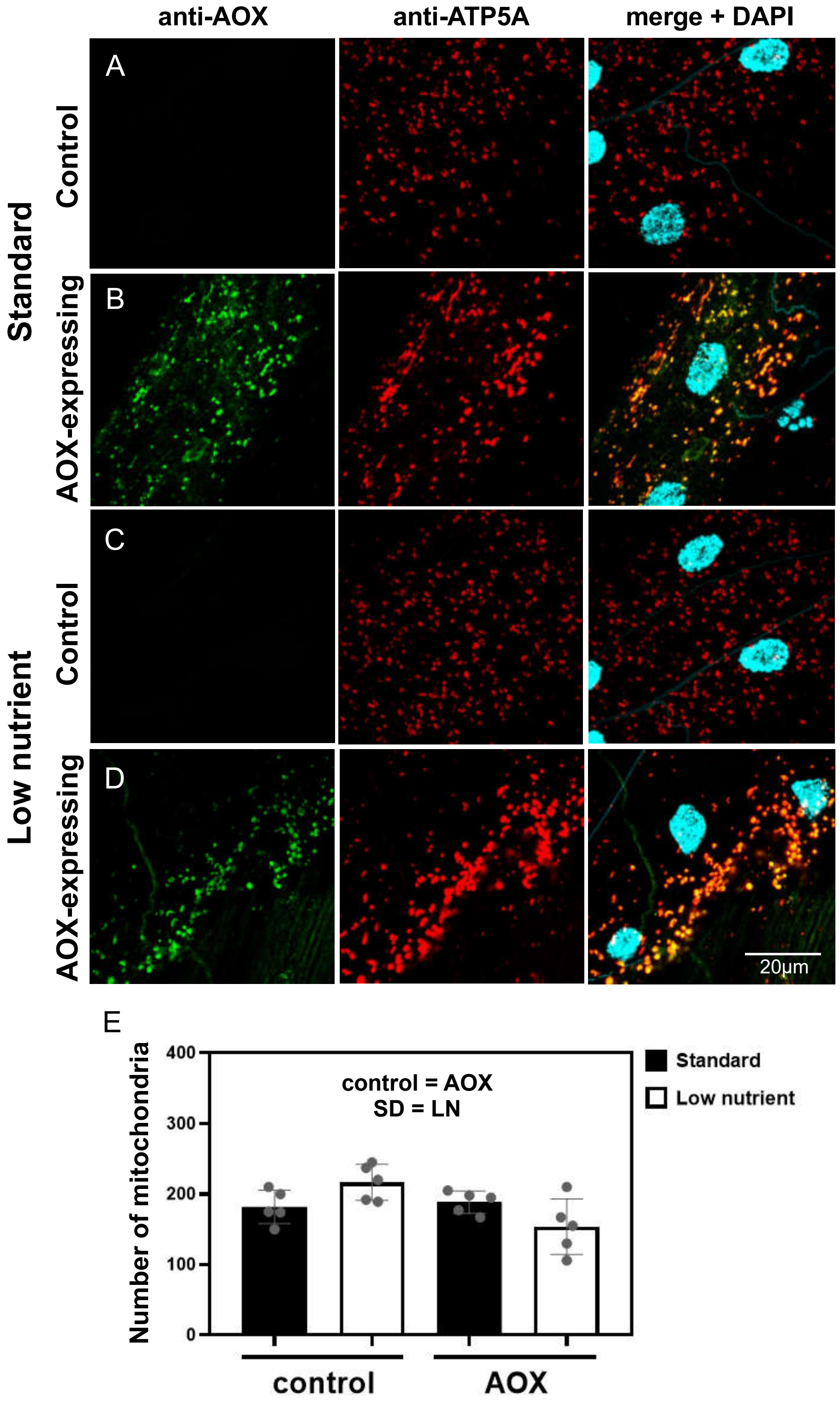
anti-AOX
anti-ATP5A
merge + DAPI
Standard
Control
AOX-expressing
Low nutrient
Control
AOX-expressing
A
B
C
D
20µm
E
Number of mitochondria
400
300
200
100
0
control = AOX
SD = LN
Standard
Low nutrient
control
AOX

Figure S2.

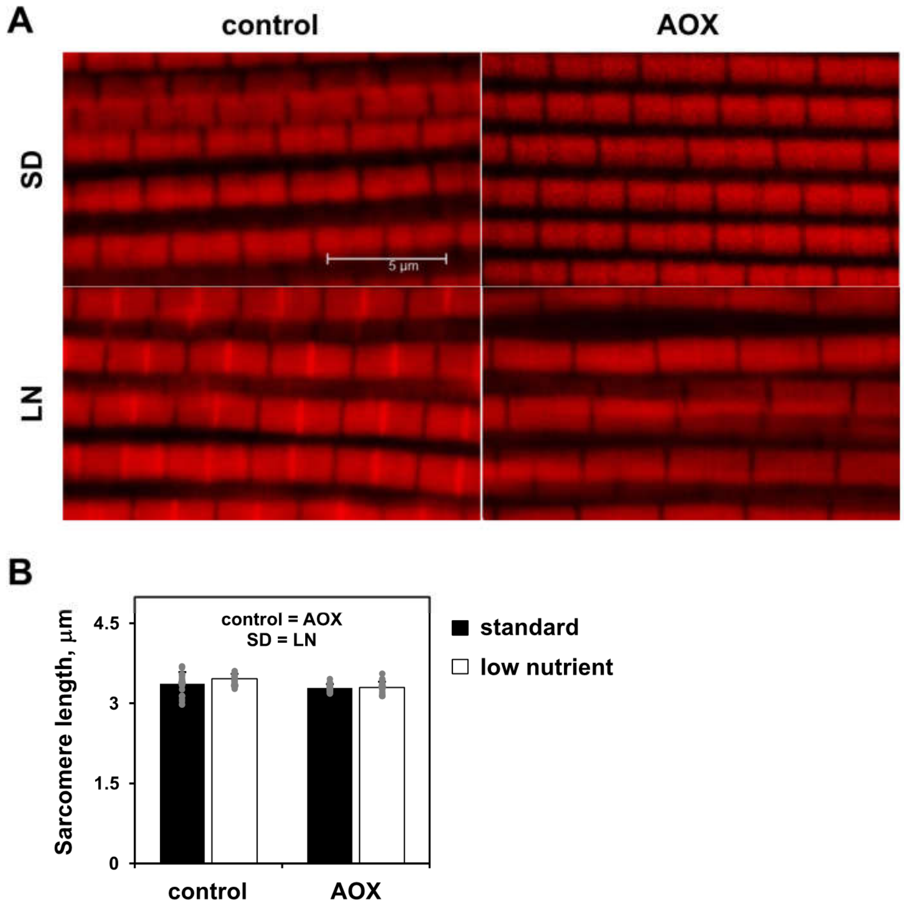

A
control
AOX
SD
LN
5 µm
B
Sarcomere length, µm
4.5
3
1.5
0
control = AOX
SD = LN
control
AOX
standard
low nutrient

Figure S3.

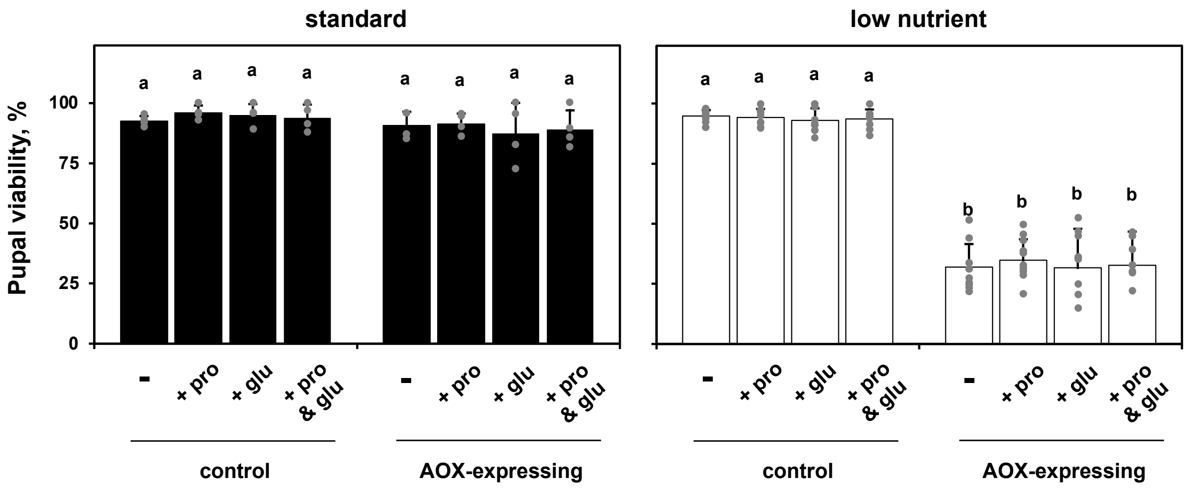
standard
low nutrient
Pupal viability, %
0
25
50
75
100
a
b
-
+ pro
+ glu
+ pro & glu
control
AOX-expressing

Figure S4.
