# Supplementary figures and images for: Impairment of Muscle Function Causes Pupal Lethality in Flies Expressing the Mitochondrial Alternative Oxidase
Source: Biomolecules. 2025 Apr 11;15(4):570. doi: 10.3390/biom15040570 (PMC12024792; doi:10.3390/biom15040570)

Blots from Figure 1B

Exposure 1

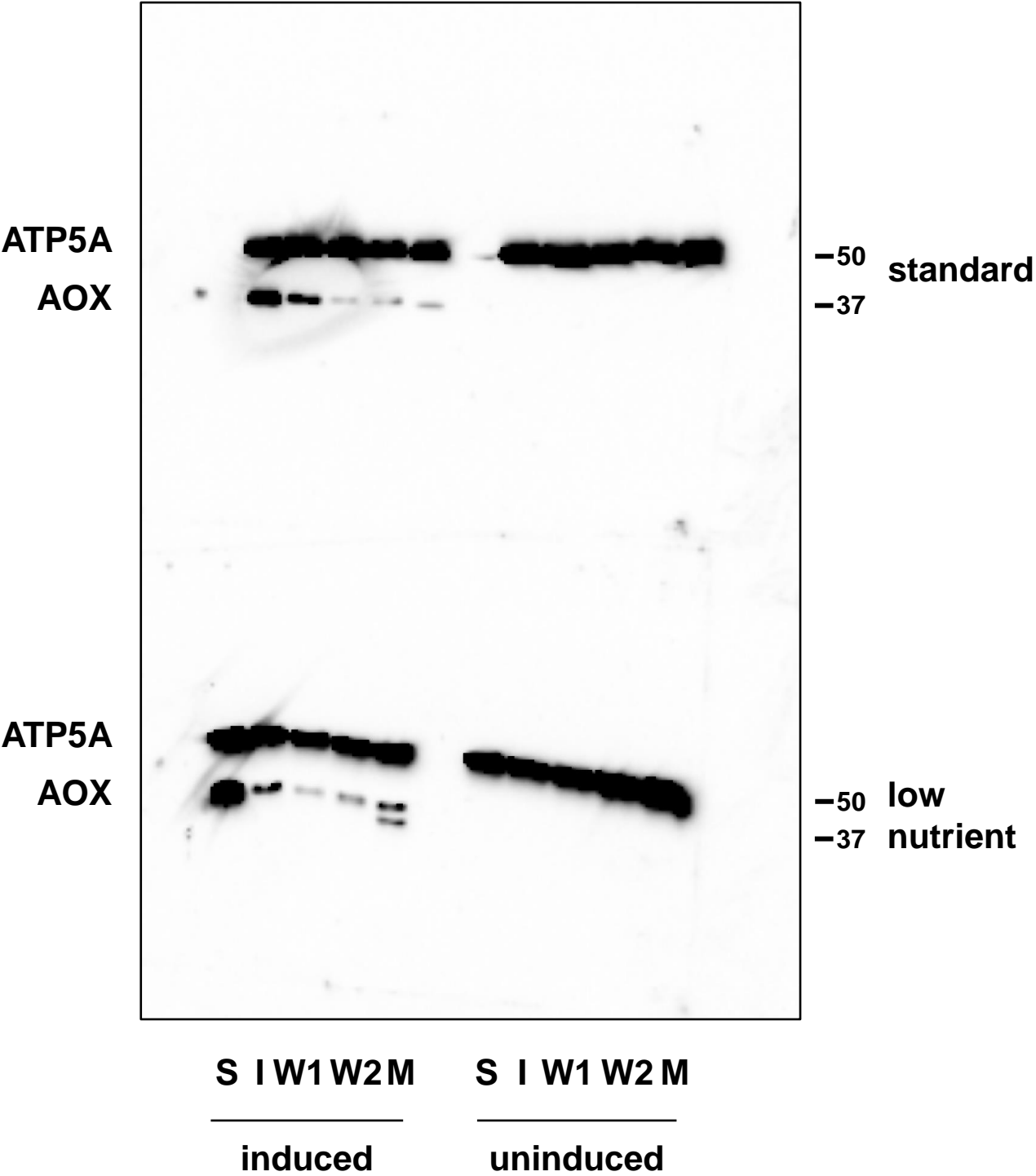

Exposure 2

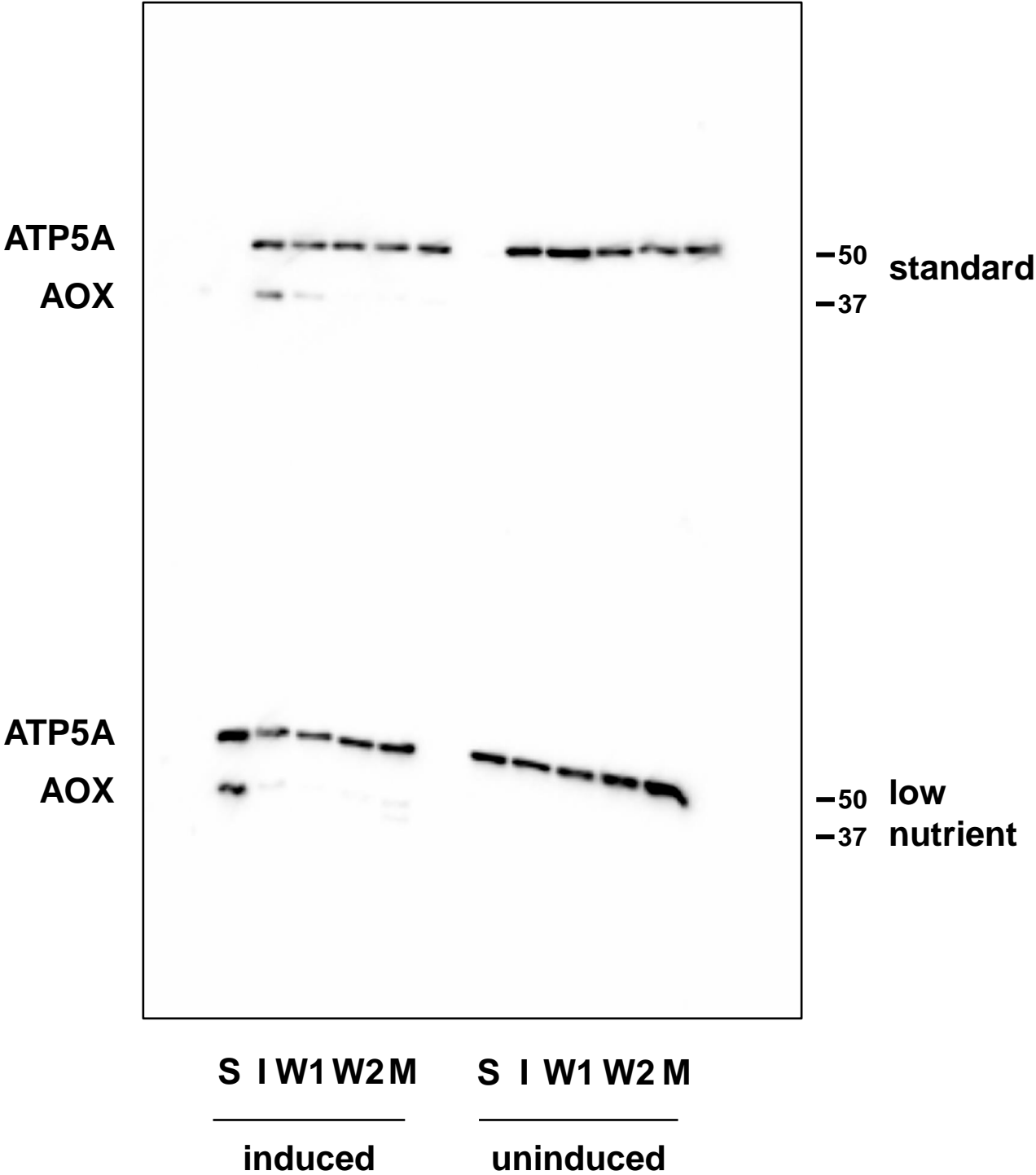

Supplement: Supplementary file 1 [file biomolecules-15-00570-s001.zip › biomolecules-3456365-original-images.pdf]
